# Relationship between weight‐carrying capacity and performance in a standardized treadmill exercise test in horses

**DOI:** 10.14814/phy2.70607

**Published:** 2025-10-07

**Authors:** Denise Söderroos, Guðrún Jóhanna Stefánsdóttir, Sveinn Ragnarsson, Víkingur Gunnarsson, Anna Jansson

**Affiliations:** ^1^ Department of Animal Biosciences, Faculty of Veterinary Medicine and Animal Science Swedish University of Agricultural Sciences Uppsala Sweden; ^2^ Department of Equine Science Hólar University Sauðárkrókur Iceland

**Keywords:** anaerobic threshold, equine, exercise physiology, rider weight, treadmill

## Abstract

Weight‐carrying capacity is important in riding horses both for performance and welfare, yet there is no standardized method to estimate individual horses' weight‐carrying capacity. This study investigated the correlation between the physiological response during a (i) standardized incremental exercise test (SET) on a treadmill and a (ii) ridden incremental weight‐carrying exercise test (WET). Sixteen horses (15 ± 3 years) performed both tests, including four steps with increased speed or weight load, respectively. Body weight ratio (BWR) in the WET was 20%, 25%, 30%, and 35% in each step, respectively. Blood samples were collected after each step, and heart rate (HR) was recorded. The velocity (SET) and BWR (WET) at a HR of 180 and 190 bpm and plasma lactate concentration of 2, 3, and 4 mmol/L were estimated. There was a correlation (*r* = 0.92, *p* < 0.05) between the velocity at a plasma lactate concentration of 3 mmol/L (V_La3_) in the SET and the BWR at a HR of 180 bpm (BWR_180_) in the WET, but no other correlations were found. In conclusion, the SET was not applicable to estimate weight‐carrying capacity in the horse. Further studies should investigate the importance of the correlation between V_La3_ and BWR_180_.

## 1 INTRODUCTION

Rider weight may impair the performance (Dyson et al., 2019; Gunnarsson et al., 2017; Stefánsdóttir, Gunnarsson, Roepstorff, et al., 2017) and influence the behavior of the horse (Dyson et al., 2019); thus, a great weight‐carrying capacity is of significant importance in riding horses. It is frequently discussed in the horse industry how heavy the rider can be, especially on Icelandic horses, and some riding schools and tour riding companies have introduced a weight limit for the rider (Douglas et al., 2022; Stefánsdóttir, Gunnarsson, Roepstorff, et al., 2017). Knowledge about assessing weight‐carrying capacity in individual horses, easily and standardized, could be useful, for example, for riding schools and other companies supplying riding services.

The effect of weight carrying has earlier been examined with different types of ridden weight‐carrying exercise tests on several horse breeds (Christensen et al., 2020; Dyson et al., 2019; Gunnarsson et al., 2017; Matsuura et al., 2016; Matsuura, Irimajiri, et al., 2013; Matsuura, Sakuma, et al., 2013; Powell et al., 2008; Stefánsdóttir, Gunnarsson, Roepstorff, et al., 2017; Van Oldruitenborgh‐Oosterbaan et al., 1995). In addition to these studies, Van Oldruitenborgh‐Oosterbaan et al. (1995) and McKeever et al. (2005) examined the effect of weights mounted on horses using a treadmill. In those studies, weight‐carrying capacity was evaluated by the physiological response (Christensen et al., 2020; McKeever et al., 2005; Powell et al., 2008; Stefánsdóttir, Gunnarsson, Roepstorff, et al., 2017; Van Oldruitenborgh‐Oosterbaan et al., 1995), evaluation of muscle soreness (Powell et al., 2008), locomotion analysis (Dyson et al., 2019; Gunnarsson et al., 2017; Matsuura et al., 2016; Matsuura, Irimajiri, et al., 2013; Matsuura, Sakuma, et al., 2013; Van Oldruitenborgh‐Oosterbaan et al., 1995), and behavior assessment (Christensen et al., 2020; Dyson et al., 2019). However, there is no “gold standard” method to estimate weight‐carrying capacity in horses. Furthermore, exercise tests with added weights require access to weights and that they are performed under standardized conditions (regarding physical environment, speed, distance, etc.). Adding a large amount of weight to the rider and/or the horse could also be a safety issue and a potential injury risk. Therefore, a standardized and safe method to estimate weight‐carrying capacity in individual horses that can be implemented worldwide is of interest. The method could, for instance, be used by riding schools and tour riding companies where there is a large variation in rider weight, but also to describe weight‐carrying capacity in individual horses for sale. Anecdotally, the conformation of a horse is often considered when estimating weight‐carrying capacity, and a few studies have evaluated the effect of conformation on weight‐carrying capacity (Powell et al., 2008; Söderroos et al., 2025; Stefánsdóttir, Gunnarsson, Roepstorff, et al., 2017). However, the evidence of the associations between conformation traits and weight‐carrying capacity is weak. With today's knowledge, estimating weight‐carrying capacity only by the conformation is unreliable.

Standardized incremental treadmill exercise tests (SETs) are commonly used to evaluate the fitness of the horse by, for example, estimating the anaerobic threshold (V_La4_) (Bitschnau et al., 2010; Harkins et al., 1993; Jansson et al., 2021). The estimated V_La4_ (either on a treadmill or at field conditions) has been shown to correlate with true performance in both Standardbred trotters (Leleu et al., 2005; Ringmark et al., 2017) and Warmblood horses (Bitschnau et al., 2010). However, it is not known if the general aerobic capacity in a horse is associated with weight‐carrying capacity and if it is possible to estimate weight‐carrying capacity with a SET without any additional weight. In humans, it has been shown that aerobic fitness (estimated with a “beep” test) is correlated with weight‐carrying capacity (Robinson et al., 2018), and the theory is that the same applies to horses.

The study aimed to investigate if the estimation of physiological parameters (e.g., V_La4_) from an incremental SET on a treadmill could be used to estimate weight‐carrying capacity in horses. The hypothesis was that the physiological response during a SET is associated with weight‐carrying capacity.

## 2 MATERIALS AND METHODS

The study was conducted at Hólar University, Iceland, in October 2021 and 2022. All horses performed two exercise tests: an incremental SET on a treadmill and a ridden incremental weight‐carrying exercise test (WET).

The study was carried out under Reg. 460/2017 on the protection of animals used for scientific purposes, as permitted by the Icelandic Food and Veterinary Authority, Ref. No. 2109502.

### 2.1 Horses and preparation

**FIGURE 1:**
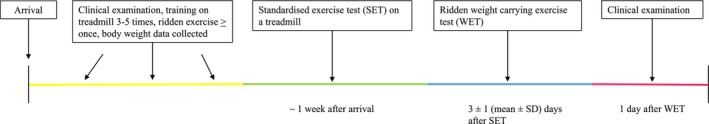
The timeline of the experiment, including two different exercise tests (SET and WET) and a preparation period.

In total, 16 Icelandic horses (seven mares and nine geldings) aged 15 ± 3 years (range 10–22 years), BW 386 ± 32 kg (range 339–452 kg), were included in the study (10 in 2021 and 6 in 2022). The number of individuals was estimated with a power calculation, with a 5% significance level, 80% power, and an expected correlation coefficient of 0.7, based on previous results (Robinson et al., 2018). The horses were owned by tour riding companies located near the University. All horses had been actively used in tours the previous summer (and occasionally in September) and were estimated by their owners to be fit enough to perform the planned exercise tests. Approximately 1 week before the experiment started, horses arrived at the University for acclimation and training (Figure 1). The horses were stabled in individual boxes and kept outside in a gravel paddock for 0–3 h/day and on pasture for 1–3 h/day. Hay (4.7 ± 0.8 MJ/kg net energy and 155 ± 26 g/kg DM crude protein) was fed three times/day in the boxes. The daily amount was individually adjusted (0.75–1.00 kg DM/100 kg BW) based on recommendations from National Research Council (2007) regarding the minimum forage intake. Horses had free water access from automatic water bowls and 2 kg salt blocks in the boxes. No feed was offered within 3 h before the exercise tests.

At least once during the preparation period, horses were ridden by the same rider that rode them in the WET in order to get used to the horses. All horses were trained on the treadmill (Säto, Lövstabruk, Sweden) at a submaximal intensity 3–5 times before the SET to acclimate to the treadmill. The treadmill training was performed in walk and trot (although some horses were occasionally tölting), including periods with uphill inclination (6.25%) of the treadmill. During the last training session on the treadmill, horses were equipped with a heart rate (HR) monitor (Polar Vantage M; Polar Electro Oy, Kempele, Finland), and the speed where the individual horse reached or exceeded a HR of ~200 bpm was noted and applied in the last step of this individual's SET. It has been reported that the speed at which HR 200 bpm is reached is close to the anaerobic threshold (Persson et al., 1991).

Prior to the SET, horses were clinically examined by a veterinarian, including auscultation of heart sounds, palpation of the limbs and the thoracolumbosacral region, and a flexion test with a trot up on a gravel road. A similar examination was performed ~24 h after the WET (Figure 1).

### 2.2 Standardized treadmill exercise test

After the preparation period (~1 week after arrival), horses performed a sub‐maximal SET (Figure 1). The SET was performed on the treadmill and consisted of four steps with increasing speed (individual basis, see above) for each step (Jansson et al., 2002, 2021; Nyman et al., 2002; Persson, 1967; Persson et al., 1991) (Table 1). The SET was preceded by a 4‐min warm‐up in walk on the treadmill before the immediate start of step 1. Each exercise step lasted for 1.5 min before the speed was increased for the next step. In one horse, the last step was only 45 s (due to refusal to go forward). The test was aimed to be performed in trot, but some horses occasionally performed steps in tölt and two horses performed step 4 in canter. The SET was finished with a 2‐min walk and 3 min of standing on the treadmill. The speed was manually determined during all steps in the SET by dividing the distance (belt length × number of revolutions) by the time measured for four revolutions. Immediately after both SET and WET, the lactate concentration in blood collected at step 4 (see below) was measured using a Lactate Pro 2 analyzer (Arkray, Kyoto, Japan). Horses with concentrations <3 mmol/L were re‐tested the day after (SET, *n* = 3), and data from the second test were used in statistical analysis.

**TABLE 1:** Description of the standardized treadmill exercise test (SET) performed in trot, including velocity (m/s) and duration (min) for each activity.

Activity	Velocity (m/s, mean ± SD)	Duration (min)
Warm‐up walk		4
Step 1^a^	3.4 ± 0.3	1.5
Step 2^a^	4.3 ± 0.4	1.5
Step 3^a^	5.1 ± 0.4	1.5
Step 4^a^	6.0 ± 0.4	1.5
Walk	1.7 ± 0.0	2
Standing		3

^a^ With a 6.25% incline.

The ambient temperature in the room during the SET was 13.0 ± 0.5°C.

### 2.3 Ridden incremental weight‐carrying exercise test

A WET was performed outside on an oval gravel track (257 m) 3 ± 1 days after the SET (Figure 1). One male professional rider and riding teacher (73 kg, 178 cm) rode horses tested in 2021, and one female riding teacher (70 kg, 165 cm) rode the horses in 2022.

The test was preceded by a mounted warm‐up (1.6 ± 0.1 km), including walk (1.5 to 2.1 m/s) for 5–7 min and 1.1 ± 0.1 km tölt (5.3 ± 0.3 m/s). The WET (total distance 2.9 ± 0.1 km, including short distances of walk to get to the predetermined start position of the test) consisted of four steps in tölt (5.7 ± 0.2 m/s, ~640 m/step, duration = ~1.9 min/step) with increasing weight carrying in each step. The WET was ridden on the left hand in all horses. The time between markings on the track was recorded using a stopwatch to facilitate speed adjustment. The rider was regularly informed via wireless communication (earpiece) if the intended speed (~5.5 m/s) was kept. A higher speed was recorded in step 4 (5.8 ± 0.1 m/s) compared with the other steps (5.6–5.7 ± 0.1 m/s), and a lower speed was recorded in step 1 (5.6 ± 0.1 m/s) than in step 2 (5.7 ± 0.1 m/s) and step 3 (5.7 ± 0.1 m/s) (*p* < 0.05). Horses carried in average 20.5 ± 1.4% (BWR_20%_), 25.3 ± 0.4% (BWR_25%_), 30.3 ± 0.3% (BWR_30%_), and 35.4 ± 0.6% (BWR_35%_) of their BW at the four steps, respectively. After the warm‐up and between each step, horses were stopped, and the rider dismounted for 5.4 ± 1.1 min to add weights and collect blood samples. Two horses were retested 2 days after the WET due to low blood lactate concentrations (~<3 mmol/L) at step 4.

Two different saddles (6 kg and 8.4 kg, respectively) were used in the test, depending on the intended weight load. The heavier saddle (Ástund Super; Ástund, Reykjavík, Iceland) had bags attached to the flaps where it was possible to add lead weights (up to 8 kg on each side). To increase the weight further, two saddle pads (13.5 kg and 20 kg, respectively), extra heavy stirrups (4 kg each), and a weight vest designed for scuba diving, filled with lead weights (up to 16 kg) inside pockets, could also be added. Due to discomfort for the rider, a weight vest specially designed for CrossFit® was used instead of the scuba diving vest during the experiment in 2022. Weights were evenly added on both sides to avoid imbalance. In three horses at the last step, the rider also carried a backpack filled with sand (up to 16 kg), either on the back or in the front. Another three horses had boots on the front hooves (155 g–170 g each) to keep a clear‐beat tölt.

Ambient temperature and wind speed were registered with an automatic weather station, located ~100 m from the riding track and were noted when the horse left the stable for the WET. In the middle of each experimental day (10 am‐13 am), the ambient temperature was 3.1 ± 4.5°C, and the wind speed was 3.6 ± 3.3 m/s. Registrations of wind speed were missing for 1 day, but it was not unusually windy that day.

### 2.4 Data collection

During both of the exercise tests, HR was measured continuously with a HR recorder (Polar Vantage M; Polar Electro Oy, Kempele, Finland). Velocity was also measured with the HR recorder (GPS) during the WET. Recordings from each step during the exercise tests were exported to Microsoft Excel 2016 (Microsoft, Redmond, WA, USA), and the mean velocity in each step was calculated, as well as the mean HR at the last 15 s in each step.

Rectal temperature (RT) was measured with a digital thermometer (Omron; HealthCare, Europe) before the SET and WET and was 37.3 ± 0.3°C and 37.1 ± 0.4°C, respectively.

### 2.5 Blood sampling and analyses

Before entering the treadmill (20–45 min prior to the exercise test), a catheter was placed in the jugular vein, and blood was drawn at the end of each step during the SET. In the WET, blood was collected by venipuncture after each step in the exercise test. Local anesthesia (20 mg/mL, Xylocaine; AstraZeneca, Södertälje, Sweden) was given before catheterisation and venipuncture. After sampling, blood was directly placed on ice and later analyzed for hematocrit (8 min, 21,913 × g, Cellokrit, AB Lars Ljungberg & Co, Stockholm, Sweden). Duplicate analyses were performed, and a mean value was used for the statistical analysis. The hematocrit value for one sample was missing in step 4 at the SET. Plasma was separated by centrifugation (10 min, 3000 rounds/min, Hettich, Tuttlingen, Germany) and stored at −18°C until lactate analysis. Plasma lactate was analyzed (YSI 2500 Biochemistry Analyzer, YSI, Yellow Springs, Ohio, USA, CV = 1.6%) in duplicate, and a mean value was used for statistical analysis.

### 2.6 Statistical analysis and calculations

Velocity at a HR of 180 bpm (V_180_) and 190 bpm (V_190_) was estimated for individual horses in Microsoft Excel 2016 (Microsoft, Redmond, WA, USA) by applying the following equation and linear relationship between velocity and mean HR of the last 15 s in each step in the SET:

y=a+bx,

where *y* is the HR (180 or 190 bpm), *a* is the HR at a velocity of 0 m/s, *b* is the slope, and *x* is the velocity at *y* = 180 or 190 bpm.

A similar procedure was performed to estimate BWR_180_ and BWR_190_ (BWR at a HR of 180 and 190 bpm, respectively) in the WET. Also, V_200_ and BWR_200_ were determined but not statistically analyzed due to few observations (i.e., several horses did not reach a HR of 200 bpm during the WET (*n* = 10) and the SET (*n* = 4), respectively).

The velocity at the treadmill where the horses reached a plasma lactate concentration of 2, 3, and 4 mmol/L (V_La2_, V_La3_, and V_La4_, respectively) was estimated by obtaining the following equation of the exponential relationship between velocity and plasma lactate concentration for each step in the SET:

$$y=a\times e^{\mathrm{bx}}$$

where *y* is the plasma lactate concentration (2, 3 or 4 mmol/L), *a* is the lactate concentration at a velocity of 0 m/s, *b* is the slope, and *x* is the velocity at *y* = 2, 3 or 4 mmol/L.

The same procedure was applied to estimate the BWR when the horse reached a plasma lactate concentration of 2, 3, and 4 mmol/L (BWR_La2_, BWR_La3_, and BWR_La4_, respectively) during the WET. Data was missing in cases where horses had a HR > 180, 190, or 200 bpm or a plasma lactate concentration >2, 3 or 4 mmol/L already at the first step or when these values were not reached during the tests (see Table 2 for *n*). In addition, data was missing for one horse with no linear HR increase during the WET.

**TABLE 2:** Physiological parameters recorded during an incremental standardized exercise test (SET) on an inclined (6.25%) treadmill and a ridden incremental weight‐carrying exercise test on a track (WET) in 16 Icelandic horses.

SET			WET		
Parameter	*n*	Mean ± SD	Parameter	*n*	Mean ± SD
V_180_ ^a^ (m/s)	14	4.7 ± 0.5	BWR_180_ ^a^ (%)	5	26.2 ± 3.4
V_190_ ^b^ (m/s)	14	5.3 ± 0.5	BWR_190_ ^b^ (%)	5	26.8 ± 4.7
V_La2_ ^c^ (m/s)	14	4.4 ± 0.4	BWR_La2_ ^c^ (%)	9	24.6 ± 3.0
V_La3_ ^d^ (m/s)	14	5.2 ± 0.5	BWR_La3_ ^d^ (%)	12	28.4 ± 4.2
V_La4_ ^e^ (m/s)	9	5.5 ± 0.5	BWR_La4_ ^e^ (%)	10	31.4 ± 4.1
Hematocrit_step4_ ^f^ (%)	15	46 ± 4	Hematocrit_35%_ ^f^ (%)	16	46 ± 2

^a^ Velocity or body weight ratio, that is, rider weight as a percentage of the horse's body weight (BWR) at a heart rate (HR) of 180 bpm.

^b^ Velocity or BWR at a HR of 190 bpm.

^c^ Velocity or BWR at a plasma lactate concentration of 2 mmol/L.

^d^ Velocity or BWR at a plasma lactate concentration of 3 mmol/L.

^e^ Velocity or BWR at a plasma lactate concentration of 4 mmol/L.

^f^ Analyzed from samples collected at step 4/BWR_35%_.

Principal component analysis (PCA) (Abdi & Williams, 2010) was performed in Umetrics SIMCA (version 17.0.2; Sartorius, Goettingen, Germany) and other statistical analyses in SAS (version 9.4; SAS Institute Inc., Cary NC, USA).

The relationship between physiological parameters recorded at the SET and WET was evaluated using Pearson's correlation test and visualization of a loadings plot for a PCA model. Data were scaled to unit variances before the PCA (Groth et al., 2013).

Possible differences in velocity between steps in the WET were analyzed with a general linear mixed model (PROC MIXED):

$$Y=\mu +a_{i}+b_{j}+e_{\mathrm{ij}}$$

where *Y* is the observation, *μ* is the mean value, *a*_*i*_ is the fixed effect of step, *b*_*j*_ is the random effect of horse, and *e*_*ij*_ is the residuals.

Normality of residuals was evaluated by observing qq‐plots. Data are presented as mean ± SD and the velocity at the different steps in the WET as LSM ± SE.

## 3 RESULTS

### 3.1 Correlations between the physiological response at the standardized treadmill exercise test and the ridden incremental weight‐carrying test

**FIGURE 2:**
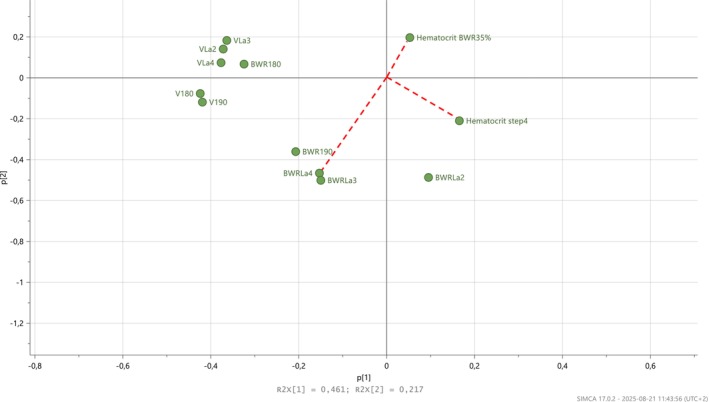
Results of a principal component analysis (PCA) that visualizes correlations between variables collected at an incremental standardized treadmill exercise test (SET) and at a ridden incremental weight‐carrying exercise test on a track (WET) in 16 Icelandic horses. Variables close to each other are positively correlated, for example, velocity (SET) at a heart rate of 180 bpm (V180) and 190 bpm (V190). Variables opposite each other, forming an angle near 180 degrees, for example, hematocrit analyzed from samples collected at step 4 in the WET (BWR35%) and body weight ratio at the anaerobic threshold in the WET (BWRLa4), are negatively correlated. Variables situated close to 90 degrees from each other, for example, hematocrit analyzed from samples collected at step 4 in the SET (Hematocrit step4) and the WET (Hematocrit 35%), are unlikely to be correlated.

**FIGURE 3:**
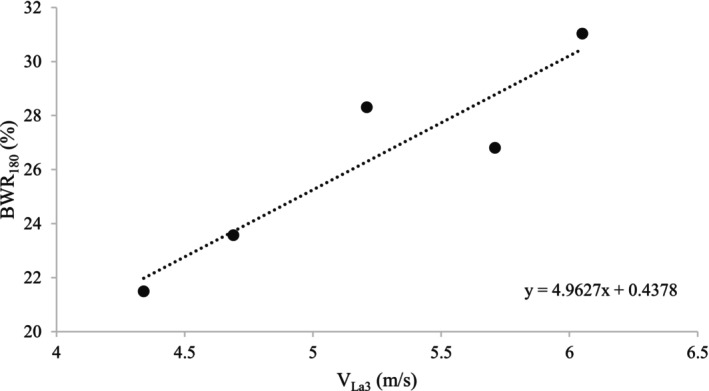
The linear relationship between the velocity at a plasma lactate concentration of 3 mmol/L in an incremental standardized treadmill exercise test (SET) and body weight ratio (BWR) at a heart rate (HR) of 180 bpm in a ridden incremental weight‐carrying exercise test on a track (WET) in Icelandic horses (*r* = 0.92, *p* < 0.05, Pearson's correlation test).

Mean values of physiological parameters recorded at the SET and WET are presented in Table 2, and a visualization of the relationships between all recorded physiological parameters (presented as loadings) during the exercise tests is shown in Figure 2. No correlations between the corresponding physiological parameters recorded at the SET and WET were found (Table 3, *p* > 0.05). According to the loadings scatter plot for the PCA model, BWR_180_ seemed to be positively correlated with V_La2_, V_La3_, and V_La4_ (Figure 2), but only the correlation between V_La3_ and BWR_180_ was significant (Pearson's correlation test, Figure 3, *r* = 0.92, *p* < 0.05). No other combination of the physiological parameters was correlated (*p* > 0.05).

**TABLE 3:** Correlations (*r*) between physiological parameters recorded during an incremental standardized exercise test (SET) on an inclined treadmill and a ridden incremental weight‐carrying exercise test on a track (WET) in 16 horses.

Physiological parameter (SET)	Physiological parameter (WET)	*n*	Correlation coefficient (*r*)	*p* Value
V_190_ ^a^	BWR_190_ ^a^	5	0.84	0.07
V_La2_ ^b^	BWR_La2_ ^b^	7	−0.40	0.37
V_La3_ ^c^	BWR_La3_ ^c^	10	0.17	0.63
V_La4_ ^d^	BWR_La4_ ^d^	5	−0.04	0.94

^a^ Velocity or body weight ratio (BWR) at a heart rate of 190 bpm.

^b^ Velocity or BWR at a plasma lactate concentration of 2 mmol/L.

^c^ Velocity or BWR at a plasma lactate concentration of 3 mmol/L.

^d^ Velocity or BWR at a plasma lactate concentration of 4 mmol/L.

## 4 DISCUSSION

This is the first study examining the association between aerobic and weight‐carrying capacity in the horse by performing two different exercise tests. There was no correlation between V_La4_ and BWR_La4_ (i.e., the anaerobic threshold for each test) or other corresponding physiological parameters recorded at the SET and the WET. However, there was a strong correlation between V_La3_ and BWR_180_. The reason for and the importance of the correlation between the lactate response at the SET and the HR response at the WET are unclear and need further investigation.

The physiological response to the two exercise tests varied among the horses, where some horses showed a relatively low physiological response even at the last step. On the contrary, some horses showed a relatively high physiological response already at the first step in the exercise tests. For example, several horses already had a HR > 180 or 190 bpm at the first step (particularly in the WET), where one horse exceeded 200 bpm. In contrast, several horses did not reach the anaerobic threshold and/or a HR of 200 bpm, not even at the last step in the exercise tests. Interestingly, horses that did not reach 200 bpm, neither at the SET nor at the WET (*n* = 3), were all 15 years or older. This could mean that they were not able to reach a HR of 200 bpm, at least not the horses that were older than 17 years, due to the age‐dependent decline in peak (Stefánsdóttir, Gunnarsson, Ragnarsson, & Jansson, 2017) and maximum HR (Betros et al., 2002). A potential age effect is important to consider in future studies evaluating the response to different exercise tests. For one horse, a linear increase of HR was not observed in the WET, and it was hypothesized that the maximum HR (or close to it) was already reached during the first step. Accordingly, several horses had missing data and correlations between V_180_ and BWR_180_ and between V_200_ and BWR_200_ were not determined in this study due to few observations (*n* < 5). In addition, the correlation between V_La4_ and BWR_La4_ could only be analyzed for five horses. However, for all horses except four (where three had technical or experimental problems), at least one of the corresponding physiological parameters (e.g., V_La4_ and BWR_La4_) could be analyzed. Furthermore, it was generally not the same horses that did not reach the anaerobic threshold during the SET and the WET, respectively. One of the horses that did not reach the anaerobic threshold during the WET had, in contrast, already reached it at the first step in the SET, which illustrates the lack of correlation between the corresponding physiological parameters. With the exception of the potential effect of high age that precludes observations at a HR of 200 bpm, the missing data was not considered to be biased.

In order to increase the number of horses that physiological parameters can be estimated for in future studies, some adjustments of the intensity of the exercise tests might be required. A lower intensity at the beginning of the tests (e.g., start with BWR < 20%) and a greater intensity at the end (BWR > 35%) will most likely increase the number of physiological parameters that can be estimated. However, from a practical perspective, it can be challenging to find a rider with a BW low enough to get a BWR < 20%, and it requires a lot of added weights at the end of the test, which highlights the difficulties estimating weight‐carrying capacity with a field test. Furthermore, younger horses than those included in the current study (age 15 ± 3 years, range 10–22 years) should preferably be studied.

The limited number of observations was certainly a weakness of the study. However, it is of interest to develop a standardized method to assess the weight‐carrying capacity on an individual basis. Therefore, if correlations are weak, an increased number of horses studied might not have been relevant.

According to the results of this study, it is not clear that weight‐carrying capacity is associated with general aerobic capacity in the horse. A SET is typically used to estimate the aerobic capacity in the unmounted horse (Seeherman & Morris, 1990; Harkins et al., 1993; Marc et al., 2000; Bitschnau et al., 2010; Jansson et al., 2021), while the WET aims to estimate the weight‐carrying capacity. In both of the tests, the physiological response is expected to increase for each step as the exercise intensity increases, either by an increase of speed (SET) or an increase of weight (WET), which was also observed (data not shown). The scientific knowledge about weight‐carrying capacity in horses and the influence of, for example, conformation or training is scarce. Horses included in this study were all used to carry riders during long‐distance tours and are expected to have a great weight‐carrying capacity. The exercise intensity during a typical tour has not been scientifically investigated but is probably relatively low considering the long distances that often are covered (Sigurðardóttir & Helgadóttir, 2015). Icelandic horses used for other purposes, such as breed evaluation field tests (BEFTs) or pace racing, are instead expected to perform during a relatively short period at a high exercise intensity (Stefánsdóttir et al., 2014; Stefánsdóttir, Gunnarsson, Ragnarsson, & Jansson, 2017). Contrary to our hypothesis, horses with great aerobic capacity might not have a great weight‐carrying capacity and vice versa. The horses in this study that had a relatively low weight‐carrying capacity, compared to the general aerobic capacity, might previously have been ridden by riders with a lower BW than those with a greater weight‐carrying capacity. It is well known that aerobic capacity can be improved with training, and the same is suggested for weight‐carrying capacity. However, it has not yet been scientifically studied in the horse. In humans, it seems like strength, power, and aerobic capacity (estimated with a “beep” test) are all important qualities for weight‐carrying capacity, with the strongest correlation between aerobic and weight‐carrying capacity (Robinson et al., 2018). However, aerobic capacity (i.e., performance in the “beep” test) was based on when the participant became exhausted and was not able to continue the test (Robinson et al., 2018) and not on objectively measured physiological parameters as in our study. Contrary to the results by Robinson et al. (2018), Bilzon et al. (2001) did not find a relationship between aerobic and weight‐carrying capacity in humans. In that study, VO_2max_ (measured at an exercise test on a treadmill) was not associated with the exercise duration of a run with 18 kg external weight. However, heavier people were able to carry the weight for a longer distance (Bilzon et al., 2001). In our study, the weight load was based on a percentage of the individual horse's BW to avoid any effect of body mass.

In addition to the theory that weight‐carrying capacity is affected by training, it may also be influenced by the conformation. Earlier studies indicate that a broad back (Stefánsdóttir, Gunnarsson, Roepstorff, et al., 2017) and loin (Powell et al., 2008) are favorable for weight‐carrying capacity. It has also been shown that body measurements in Icelandic horses used for tour riding and in riding schools and horses participating in a BEFT differ (Söderroos et al., 2024). In a recent study, a wider chest, “uphill” conformation (i.e., higher in the front than in the hind), a straight backline, and a smaller hock circumference were associated with weight‐carrying capacity in Icelandic horses, but not back shape (Söderroos et al., 2025).

As discussed above, a limitation of the current study was the substantial amount of missing data due to a great variation in physiological response among the horses. In an earlier study of weight‐carrying capacity in Icelandic horses, the mean BWR_La4_ was 23%, where the greatest value was 27.5% (Stefánsdóttir, Gunnarsson, Roepstorff, et al., 2017). Therefore, it was unexpected that several horses in the present study did not reach the anaerobic threshold, although carrying 35% of their BW. In two horses, HR was higher in step 1 than in step 2 in the SET, and the value for step 1 was therefore removed. The relatively high HR values in step 1 for these horses were hypothesized to be explained by a stress reaction despite at least three training sessions on the treadmill before the SET. King et al. (1995) have recommended at least 1–2 training sessions prior to testing, but these horses might have needed more. Similar observations were found by Jansson et al. (2021). Another weakness was that the SET and WET were performed in different gaits (trot and tölt, respectively). Tölt is often used in tours and may limit the movement of the rider in comparison to trot. Therefore, tölt was used in the WET in this study. On the other hand, trot was more practical for the SET since it was the most common gait the horses chose when running freely on the treadmill. However, we have earlier shown that there are no differences in the physiological response between tölt and trot at the velocity used in the present study (Stefánsdóttir et al., 2015).

### 4.1 Relevance for the Icelandic horse breed community

The Icelandic horse is a popular riding horse breed that is present worldwide (International Federation of Icelandic Horse Associations (FEIF), 2024) and used for various tasks. Despite their rather small size, with a mean height at withers of around 140 cm and a mean BW of 330–370 kg, they are often ridden by adult riders, some of them weighing over 100 kg (Stefánsdóttir et al., 2014). Due to their relatively small size in proportion to their riders, a great weight‐carrying capacity is especially important in these horses. Therefore, knowledge about how weight‐carrying capacity can be estimated easily and standardized in individual Icelandic horses is warranted. Such a test could be useful for, for example, tourist companies and riding schools with many horses and riders with varying BWs.

## 5 CONCLUSIONS

A strong correlation between V_La3_ and BWR_180_ was found, but no other physiological parameter measured at the SET was associated with weight‐carrying capacity. The results indicate that a SET on a treadmill, with a similar protocol as in this study, might not be applicable for estimating weight‐carrying capacity in the horse. It is still somewhat unclear if aerobic capacity is associated with weight‐carrying capacity in the horse. Further studies are needed to investigate the importance and applicability of the significant correlation between the plasma lactate response at the SET and the HR response at the WET.

## FUNDING INFORMATION

The study was supported by the Marie‐Claire Cronstedts Foundation, the Stock Protection Fund of the Icelandic Horse breed, and the Agricultural Productivity Fund of Iceland (grant number 19‐005).

## CONFLICT OF INTEREST STATEMENT

The authors declare no conflict of interest.

## ETHICS STATEMENT

The study was carried out under Reg. 460/2017 on the protection of animals used for scientific purposes, as permitted by the Icelandic Food and Veterinary Authority, Ref. No. 2109502.

## Data Availability

The datasets used and analyzed during the current study are available from the corresponding author on reasonable request.
